# Changes in Gel Characteristics, Rheological Properties, and Water Migration of PSE Meat Myofibrillar Proteins with Different Amounts of Sodium Bicarbonate

**DOI:** 10.3390/molecules27248853

**Published:** 2022-12-13

**Authors:** Zhong-Wei Wu, Xiao-Li Zou, Peng-Lei Yao, Zhuang-Li Kang, Han-Jun Ma

**Affiliations:** 1 School of Life Science and Technology, Henan Institute of Science and Technology, Xinxiang 453003, China; 2 School of Food Science, Henan Institute of Science and Technology, Xinxiang 453003, China; 3 Department of Food Testing, Luohe Vocational College of Food, Luohe 462300, China; 4 School of Tourism and Cuisine, Yangzhou University, Yangzhou 225127, China

**Keywords:** sodium bicarbonate, myofibrillar protein, texture, dynamic rheology, low-field nuclear magnetic resonance

## Abstract

The changes in the gel and rheological properties and water-holding capacity of PSE meat myofibrillar proteins with different amounts of sodium bicarbonate (SC, 0–0.6/100 g) were studied. Compared to the PSE meat myofibrillar proteins with 0/100 g SC, the texture properties and cooking yield significantly increased (*p* < 0.05) with increasing SC; meanwhile, adding SC caused the gel color to darken. All samples had similar curves with three phases, and the storage modulus (G’) values significantly increased with the increasing SC. The thermal stability of the PSE meat myofibrillar proteins was enhanced, and the G’ value at 80 °C increased with the increasing SC. Because water was bound more tightly to the protein matrix, the initial relaxation times of T_21_ and T_22_ shortened, the peak ratio of P_21_ significantly increased (*p* < 0.05), and the P_22_ significantly decreased (*p* < 0.05), which implied that the mobility of the water was reduced. Overall, SC could improve the thermal stability of the PSE meat myofibrillar proteins and increase the water-holding capacity and textural properties of the cooked PSE meat myofibrillar protein gels.

## 1. Introduction

Currently, it is important to control PSE meat during pork meat production in slaughterhouses because such meat has a poor water-holding capacity, pale color, and soft texture [1,2]. The incidence of PSE meat in China is 6–10% but higher in summer, when it is as high as 38.65% [3]. Compared to normal meat, PSE meat has the same safety and edibility, but its processing properties are inferior. Improving the processing properties of a factory is a difficult endeavor. Some studies have reported methods to solve this problem, including physical (high-pressure and ultrasonic wave), chemical (phosphate), and enzymatic (microbial transglutaminase) methods, but these are difficult to apply on a large scale in industrial production due to factors such as price, technology, and law [4,5,6].

SC is an alkali- and phosphorus-free compound and an important additive that can be added according to the product requirements in China. Some studies have reported that it has been used in meat products and other foods [7,8,9,10]. Previous studies found that substituting sodium chloride with SC can reduce cooking loss in pork and chicken batters [9,11]. Li, Zhang, Lu, and Kang [12] and Kang et al. [13] reported that when the SC content of myofibrillar proteins solution was increased from 0/100 g to 0.4/100 g, the Ca^2+^-ATPase activity and particle size significantly decreased while the pH, solubility, and active sulfhydryl and surface hydrophobicity groups significantly increased. Additionally, when the SC content was reduced from 2.0/100 g to 0.8/100 g, it led to a significantly increased salt-soluble protein concentration, water-holding capacity, b^*^ value, and storage modulus (G’) value at 80 °C in the normal pork batters. The mobility of the water was lower and the immobilized water was higher except for the 0.4/100 g SC. Our previous study reported that adding SC could elevate the pH and increase the electrostatic repulsion of PSE meat myofibrillar proteins, decrease the particle size of protein aggregates, and form more sulfhydryl and hydrophobic groups, thereby increasing the protein solubility and emulsion properties [14]. However, the effects of SC on the rheological and gel characteristics of PSE meat myofibrillar proteins have not been studied. Therefore, the aim of this research was to explore the changes in the water-holding capacity, rheological properties, and gel characteristics of PSE meat myofibrillar proteins using 0–0.6/100 g SC to obtain a novel way to improve the meat’s processing properties.

## 2. Result and Discussion

### 2.1. Cooking Yield

**Figure 1:**
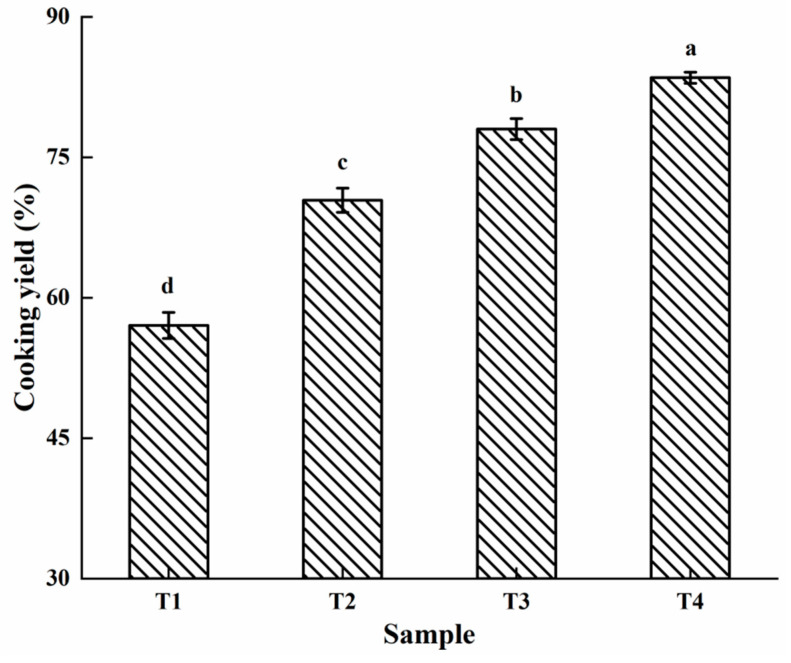
The cooking yield of the PSE meat myofibrillar proteins with varying amounts of added sodium bicarbonate. T1, 0/100 g sodium bicarbonate; T2, 0.2/100 g sodium bicarbonate; T3, 0.4/100 g sodium bicarbonate; T4, 0.6/100 g sodium bicarbonate. Each value represents the mean value ± SE (*n* = 3). ^a–d^ Different parameter superscripts in the same column indicate significant differences (*p* < 0.05).

The water retention of the gel is a vital factor that reflects the gel characteristics of myofibrillar proteins [15]. Water retention refers to the water-holding ability of proteins, while an increase in water retention indicates that water is stored in the protein gel network [16]. Figure 1 shows the effect of the SC concentrations on the cooking yield of PSE meat myofibrillar protein. With the increasing SC, the cooking yield significantly increased (*p* < 0.05); T4 showed the highest cooking yield. This indicated that the solution system with SC formed a better gel structure during the heating process, thereby improving the water-holding capacity. This was because the addition of SC increased the negative charges, which led to a pH deviation from the isoelectric point of the myofibrillar proteins due to the enhanced interaction between the protein molecules, which then enhanced the water-retention ability [14,17,18]. Lu et al. [9] reported that because SC addition improved the solubility of myofibrillar proteins, the chicken batter with SC had a higher cooking yield than that with sodium chloride. Because SC caused the structure of the chicken breast to relax, its water retention was improved in curing [19].

### 2.2. Whiteness

**Figure 2:**
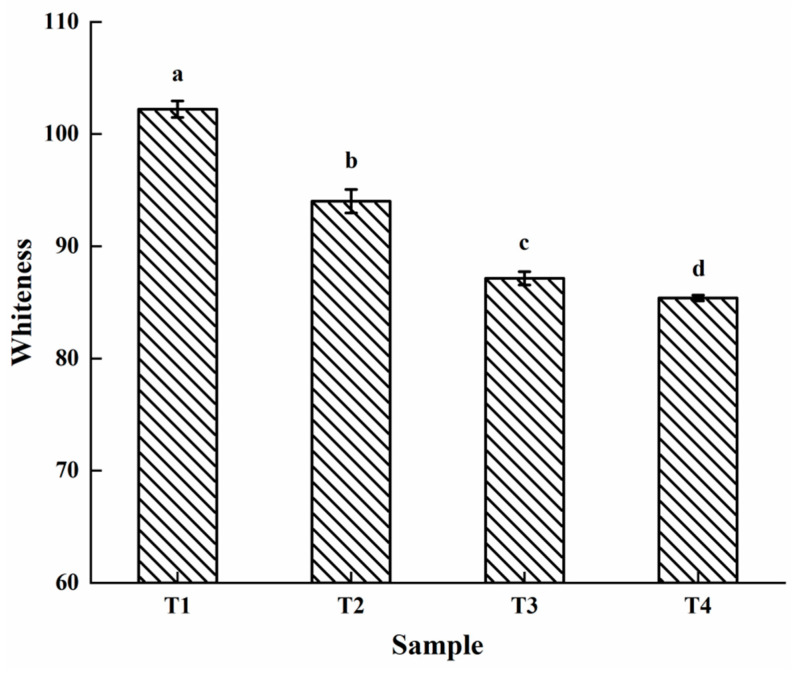
The whiteness of the PSE meat myofibrillar protein gels with varying amounts of added sodium bicarbonate. T1, 0/100 g sodium bicarbonate; T2, 0.2/100 g sodium bicarbonate; T3, 0.4/100 g sodium bicarbonate; T4, 0.6/100 g sodium bicarbonate. Each value represents the mean value ± SE (*n* = 3). ^a–d^ Different parameter superscripts in the same column indicate significant differences (*p* < 0.05).

Whiteness, which is an important indicator, is used to measure the character of meat products and directly affects consumer acceptability. As shown in Figure 2, compared to the sample with 0/100 g SC, the whiteness significantly decreased (*p* < 0.05) from 94.01 to 85.39 with the increasing SC. T4 had the lowest whiteness index value. This result indicated that the concentration of SC affected the color of the cooked PSE meat myofibrillar proteins. A previous study reported that the color of cooked chicken breast meat batters darkened with a higher whiteness when SC was added [11]. Petracci et al. [20] showed that the color of chicken breast marinated with less than or equal to 0.3/100 g SC was significantly darker than the control group. Wachirasiri et al. [21] found that the addition of 1/100 g lysine and 1/100 g SC significantly affected the color of white shrimp. Additionally, some studies reported that SC reduced myoglobin denaturation during cooking and darkened the color of meat [20,22,23].

### 2.3. Textural Properties

Texture is a vital index that reflects the quality of heat-induced myofibrillar protein gels [24]. Table 1 shows the effects of SC concentrations on the textural properties of the cooked PSE meat myofibrillar proteins. Compared to the sample with 0/100 g SC, the hardness, springiness, cohesiveness, and chewiness of the cooked solution system significantly increased (*p* < 0.05) with the increasing SC. SC is a strong base and weak acid salt that can increase the pH and negative charge of a solution system and improve the solubility of myofibrillar proteins [13,20]. Some researchers have reported that the β-sheet structure content of muscle proteins increased with increasing SC and that the β-sheet structure was the basis of gel formation [9,11]. Bertram et al. [25] reported that SC-containing samples had a higher protein solubility, which reduced the negative effects of pork muscle protein denaturation during heating. Zou, Kang, Li, and Ma [14] reported that the solubility and proportions of free sulfhydryl and surface hydrophobicity groups of myofibrillar proteins in PSE meat significantly increased with an increasing SC concentration, thereby resulting in a better gel structure. Kang et al. [13] found that the hardness, springiness, cohesiveness, and chewiness of cooked normal pork batters increased with the addition of SC. Thus, SC could improve the textural properties of PSE meat myofibrillar protein gels.

### 2.4. Rheological Properties

**Figure 3:**
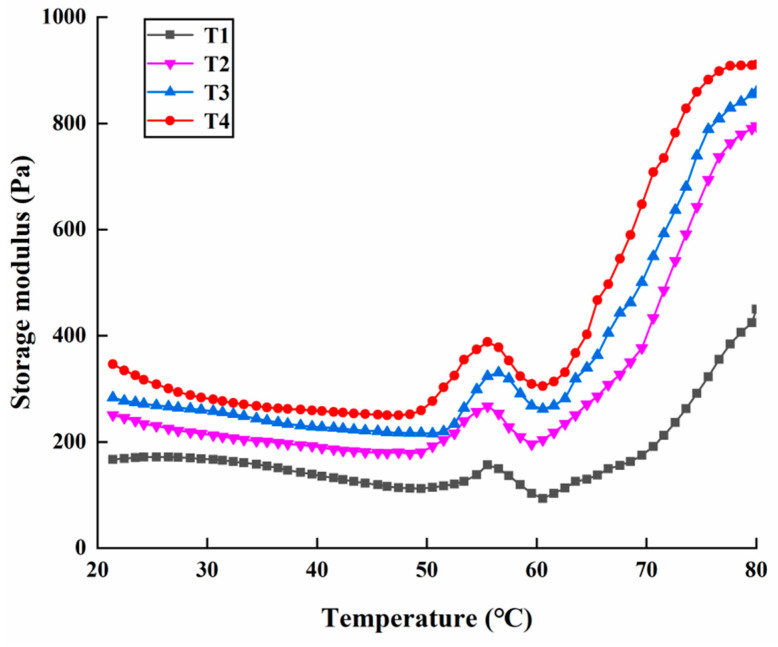
The changes in G′ values of the PSE meat myofibrillar protein gels with varying amounts of added sodium bicarbonate. T1, 0/100 g sodium bicarbonate; T2, 0.2/100 g sodium bicarbonate; T3, 0.4/100 g sodium bicarbonate; T4, 0.6/100 g sodium bicarbonate.

The change in the G’ of a sample is typically used to reflect the changes in the elasticity and viscosity of the gel [26]. Changes in the G’ of the raw solution system with varying amounts of added SC are shown in Figure 3. All samples had similar curves, and the changes in the G’ exhibited three phases. In the first phase, a slight decrease in the G’ of all samples from 20 °C to approximately 50 °C occurred. In the second phase, a rapid increase in the G’ of all the samples from approximately 50 °C to 55 °C (T1), 56 °C (T2), and 57 °C (T3 and T4) were observed, which was related to the interaction of myosin heads. Then, due to the myosin tail degeneration, a slight decrease in the G’ of all samples was observed up to approximately 60 °C [27,28]. In the third phase, the G’ values of all samples rapidly increased with the increasing temperature, indicating that myofibrillar protein aggregation occurred and that a three-dimensional network structure with good elasticity was formed [29]. Moreover, all G’ values of the sample with a high SC content were larger than those with low SC content during the heating process. T4 had the highest G’ value at 80 °C (909.25 Pa), which was 2.02 times that of T1 (449.93 Pa), implying that the elasticity of the cooked solution system significantly increased with the increasing SC, which was in agreement with the result of textural properties (Table 1).

### 2.5. LF-NMR Measurement

**Figure 4:**
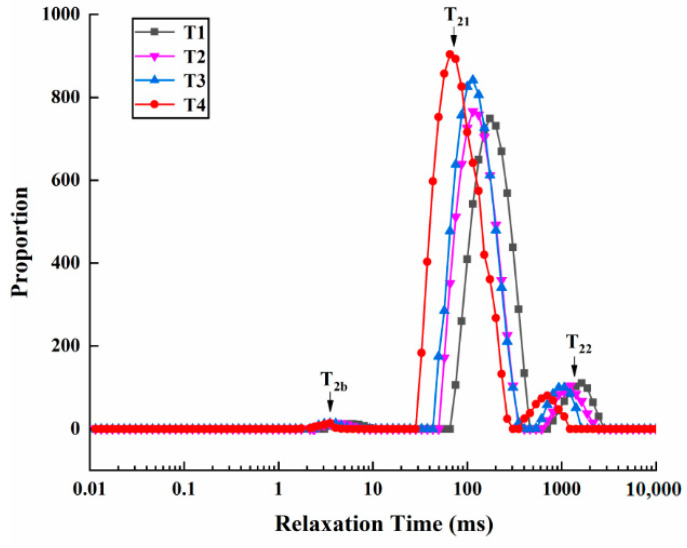
The changes in relaxation time (ms) of the PSE meat myofibrillar protein gels with varying amounts of added sodium bicarbonate. T1, 0/100 g sodium bicarbonate; T2, 0.2/100 g sodium bicarbonate; T3, 0.4/100 g sodium bicarbonate; T4, 0.6/100 g sodium bicarbonate.

It is well known that LF-NMR can reflect the water distribution in myofibrillar protein gels and that the relaxation time represents the degree of binding between the sample and water; the shorter the relaxation time, the better the retention of water [13]. Changes in the initial relaxation time and peak ratio of the cooked solution system with different amounts of SC are shown in Table 2 and Figure 4. T_2b_, T_21_, and T_22_ represent bound water, immobile water, and free water, respectively [30]. The SC content had a significant effect (*p* < 0.05) on the relaxation times of the samples. Compared with that of the T1 sample, the initial relaxation times of T_2b_ in the T2, T3, and T4 samples significantly decreased and were not significantly different (*p* > 0.05) as the SC increased. Kang et al. [13] reported that SC could increase the bound water content of cooked pork batters. Furthermore, compared with the T1 sample, the relaxation times of T_21_ and T_22_ in the T2, T3, and T4 samples significantly decreased (*p* < 0.05) with the increasing SC, indicating that the immobile water and free water were tied more tightly and that the water fluidity was lowered [31,32]. The changes in the peak ratio (P_2_) of the PSE meat myofibrillar protein gels were in agreement with the results (Figure 4).

P_2_ is typically used to evaluate water migration in myofibrillar protein gels [33,34]. The P_2b_ values in all the samples were not significantly different (*p* > 0.05). Compared with the T1 sample, P_21_ in the T2, T3, and T4 samples significantly increased (*p* < 0.05) with the increasing SC. Conversely, compared with the T1 sample, P_22_ in the T2, T3, and T4 samples significantly decreased (*p* < 0.05) with the increasing SC. Thus, T4 had the highest P_21_ and the lowest P_22_, implying that the contents of immobile water increased and free water decreased. In summary, the immobile water of the cooked solution system was bound more tightly to the protein matrix, and the mobility of the water was lowered with the increasing SC.

## 3. Materials and methods

### 3.1. Experimental Materials

The PSE chilled pork meat *longissimus lumborum* of 8 pigs (100 ± 5 kg) was purchased from a local slaughter house (Xinxiang, China) according to our previous method [14]. After grinding and vacuum packaging, the meat was stored at −80 °C. All the reagents were analytically pure.

### 3.2. Extraction of PSE Meat Myofibrillar Proteins

According to the method of Kang et al. [13], the PSE meat myofibrillar proteins were extracted from the ground meat. Briefly, the PSE meat was homogenized in four volumes of a buffer (100 mmol**/**L Tris, 10 mmol**/**L EDTA, pH 8.3) in a T25 digital homogenizer (IKA Ltd., Staufen, Germany). The homogenates were centrifuged (4 °C) at 1000× *g* for 20 min (Sorvall LYNX4000, Thermo Fisher Scientific, Langenselbold, Germany). The supernatant was removed, and the sediments were resuspended in four volumes of a buffer (100 mmol**/**L KCl, 20 mmol**/**L K_2_HPO_4_/KH_2_PO_4_, 2 mmol**/**L MgCl_2_, 1 mmol**/**L EGTA, 1 mmol**/**L NaN_3_; pH 7.0) and centrifuged at 1000× *g* for 10 min under the same conditions above again. After that, the sediments were resuspended in four volumes of another buffer (100 mmol**/**L KCl, 20 mmol**/**L K_2_HPO_4_/KH_2_PO_4_, 2 mmol**/**L MgCl_2_, 1 mmol**/**L EGTA, 0.1 g/L NaN_3_, 10 g/L Triton X-100; pH 7.0), and then centrifuged (1500× *g* for 10 min) at 4 °C. The supernatant was removed, and the sediments were resuspended in four volumes of 0.1 mol**/**L KCl solution and centrifuged at 1500× *g* for 10 min (4 °C). Next, the sediments were resuspended in four volumes of 0.1 mol**/**L NaCl solution and centrifuged at 1500× *g* for 10 min (4 °C). Finally, the purified myofibrillar protein sediment was obtained, stored at 4 °C, and used within 24 h.

### 3.3. Preparation of the Solution and Gel of PSE Meat Myofibrillar Proteins

The PSE meat myofibrillar proteins were diluted to 80 mg/mL with 50 mmol/L K_2_HPO_4_/KH_2_PO_4_ phosphate buffer and 0.26 mol/L sodium chloride (pH 6.0). Following this, 0/100 g (T1), 0.2/100 g (T2), 0.4/100 g (T3), and 0.6/100 g (T4) SC were added to the solution system, respectively. After that, the solution was uniformly homogenized (1000 rpm, 6 s) at 2 ± 2 °C.

After being left at 2 ± 2 °C for 30 min, approximately 10 g of solution was put into a beaker and heated in a water bath at 80 °C for 30 min (the core temperature was 72 °C). Finally, the cooked PSE meat myofibrillar proteins were stored at 2 ± 2 °C.

### 3.4. Cooking Yield

After being stored overnight at 2 ± 2 °C, we removed the excess water from the cooked PSE meat myofibrillar proteins and weighed. The formula was calculated for the cooking yield as follows:

Cooking yield (%) = Weight of cooked batter/Weight of raw batter × 100%(1)

All treatments were determined five times.

### 3.5. Whiteness

The whiteness of the cooked PSE meat myofibrillar proteins was determined using a colorimeter (CR-400, Minolta Camera Co., Tokyo, Japan) and calibrated with a standard white colorimeter. The formula for whiteness was calculated as follows:

Whiteness = 100 − [(100 − L*)^2^ + (a*)^2^ + (b*)^2^]^1/2^ (2)

All treatments were determined five times.

### 3.6. Textural Properties

After being stored overnight at 2 ± 2 °C, the cooked PSE meat myofibrillar proteins were left at 20 °C for 2 h. Following this, the samples were cut into cylinders (diameter: 16 mm; height: 16 mm). The textural properties of the samples (hardness, springiness, cohesiveness, and chewiness) were measured while referring to the method by Kang et al. [35]. The setting parameters were as follows: pre-test speed of 5.0 mm/s; test speed of 2.0 mm/s; post-test speed of 5.0 mm/s; strain of 50%; time of 5.0 s; and trigger force of 5 g. The values of hardness (N), springiness, adhesiveness, and chewiness (N mm) were obtained. All treatments were determined five times.

### 3.7. Dynamic Rheological Properties

The dynamic rheological properties of the 80 mg/mL raw PSE meat myofibrillar proteins were measured during heating from 20 °C to 80 °C (Haake Mars III, Thermo Scientific, Germany) while referring to the method by Zhu et al. [11]. The temperature was kept at 20 °C for 10 min then raised at a rate 2 °C/min from 20 °C to 80 °C. Continuous shearing in an oscillatory mode at a fixed frequency of 0.1 Hz was used, and the changes in the storage modulus (G’) were measured during the process. All treatments were determined four times.

### 3.8. Low-Field Nuclear Magnetic Resonance (LF-NMR)

The NMR relaxation (T_2_) measurement of the cooked PSE meat myofibrillar proteins was conducted using an NMI20-040V-1 NMR analyzer (Niumag Electric Corporation, Shanghai, China) while referring to the method by Kang et al. [36]. All treatments were determined four times.

### 3.9. Statistical Analysis

The entire experiment was repeated 3 times on different occasions (*n* = 3); the results are given as the mean ± SE. The data were analyzed using the one-way ANOVA program and the general linear model (GLM) procedure (SPSS v.18.0). Significant differences between means were identified by the LSD procedure; the results were considered significant at *p <* 0.05.

## 4. Conclusions

This study showed that SC had significant effects on the gel quality and water retention of PSE meat myofibrillar proteins. With the increasing SC, the cooking yield, hardness, springiness, cohesiveness, and chewiness of the PSE meat myofibrillar proteins significantly increased and the whiteness value significantly decreased. The results of dynamic rheology showed that the thermal stability of myosin was enhanced and the G′ value at 80 °C was increased with the increasing SC, which led to the relaxation time of T_21_ and T_22_ in the samples becoming shorter and the mobility of the water becoming slow. In sum, adding SC can improve the gel properties and water-holding capacity of PSE meat myofibrillar proteins.

## Figures and Tables

**Table 1:** The texture properties of the PSE meat myofibrillar protein gels with varying amounts of added sodium bicarbonate.

Sample	Hardness (g)	Springiness	Cohesiveness	Chewiness (g.mm)
T1	110.52 ± 7.76 ^d^	0.57 ± 0.06 ^d^	0.33 ± 0.02 ^d^	21.08 ± 2.95 ^d^
T2	141.33 ± 7.49 ^c^	0.72 ± 0.06 ^c^	0.44 ± 0.04 ^c^	48.88 ± 4.55 ^c^
T3	157.27 ± 1.52 ^b^	0.83 ± 0.03 ^b^	0.52 ± 0.02 ^b^	75.99 ± 3.01 ^b^
T4	248.43 ± 16.15 ^a^	0.91 ± 0.02 ^a^	0.57 ± 0.05 ^a^	115.36 ± 2.64 ^a^

T1, 0/100 g sodium bicarbonate; T2, 0.2/100 g sodium bicarbonate; T3, 0.4/100 g sodium bicarbonate; T4, 0.6/100 g sodium bicarbonate. Each value represents the mean value ± SE, *n* = 3. ^a–d^ Different parameter superscripts in the same column indicate significant differences (*p* < 0.05).

**Table 2:** The changes in initial relaxation time (ms) and peak ratio (%) of the PSE meat myofibrillar protein gels with varying amounts of added sodium bicarbonate.

Scheme	Initial Relaxation Time (ms)			Peak Ration (%)		
	T_2b_	T_21_	T_22_	P_2b_	P_21_	P_22_
T1	3.20 ± 0.27 ^a^	69.08 ± 5.69 ^a^	740.69 ± 61.00 ^a^	0.78 ± 0.04 ^a^	84.03 ± 2.35 ^c^	13.25 ± 2.74 ^a^
T2	2.38 ± 0.11 ^b^	54.74 ± 4.30 ^b^	586.95 ± 46.14 ^b^	0.76 ± 0.25 ^a^	87.90 ± 1.87 ^b^	9.53 ± 0.94 ^b^
T3	2.30 ± 0.10 ^b^	41.41 ± 3.26 ^c^	444.00 ± 34.90 ^c^	0.74 ± 0.01 ^a^	91.24 ± 0.16 ^a^	5.78 ± 0.33 ^c^
T4	2.19 ± 0.03 ^b^	29.90 ± 2.46 ^d^	335.87 ± 26.40 ^d^	0.66 ± 0.04 ^a^	93.65 ± 0.20 ^a^	3.04 ± 0.53 ^c^

T1, 0/100 g sodium bicarbonate; T2, 0.2/100 g sodium bicarbonate; T3, 0.4/100 g sodium bicarbonate; T4, 0.6/100 g sodium bicarbonate. Each value represents the mean value ± SE (*n* = 3). ^a–d^ Different parameter superscripts in the same column indicate significant differences (*p* < 0.05).

## Data Availability

Research data are not shared.

## References

[1] Paula M.M.D., Haddad G.D.S., Rodrigues L.M., Júnior A.A., Romos A.D.S., Ramos E.M. (2019). Effects of PSE meat and salt concentration on the technological and sensory characteristics of restructured cooked hams. Meat Sci..

[2] Xu Z.Q., Shao Y.G., Liu G.J., Xing S.J., Zhang L., Zhu M.R., Xu Y.L., Wang Z.R. (2021). Proteomics analysis as an approach to understand the formation of pale, soft, and exudative (PSE) pork. Meat Sci..

[3] Liu R., Wu G.Y., Li K.Y., Ge Q.F., Wu M.G., Yu H., Wu S.L., Bao W.B. (2021). Comparative Study on Pale, Soft and Exudative (PSE) and Red, Firm and Non-Exudative (RFN) Pork: Protein Changes during Aging and the Differential Protein Expression of the Myofibrillar Fraction at 1 h Postmortem. Foods.

[4] Li K., Kang Z.L., Zhao Y.Y., Xu X.L., Zhou G.H. (2014). Use of High-Intensity Ultrasound to Functional Properties of Batter Suspensions Prepared from PSE-like Chicken Breast Meat. Food Bioprocess Technol..

[5] Lesiow T., Rentfrow G.K., Xiong Y.L. (2017). Polyphosphate and myofibrillar protein extract promote transglutaminase-mediated enhancements of rheological and textural properties of PSE pork meat batters. Meat Sci..

[6] Bian G., Xue S., Xu Y., Xu X., Han M. (2018). Improved gelation functionalities of myofibrillar protein from pale, soft and exudative chicken breast meat by nonenzymatic glycation with glucosamine. Int. J. Food Sci. Technol..

[7] dos Santos V.M.O., Caldara F.R., de Oliveira Seno L., Feijó G.L.D., Garcia R.G., Altemio Â.D.C. (2012). Marinade with alkaline solutions for the improvement of pork quality. Pesqui. Agropecuária Bras..

[8] Murthy L.N., Phadke G.G., Jeyakumari A., Ravishankar C.N. (2020). Effect of added calcium and heat setting on gel forming and functional properties of *Sardinella fimbriata* surimi. J. Food Sci. Technol..

[9] Lu F., Kang Z.L., Wei L.P., Li Y.P. (2021). Effect of sodium bicarbonate on gel properties and protein conformation of phosphorus-free chicken meat batters. Arab. J. Chem..

[10] Kang Z., Shang X., Li Y., Ma H. (2022). Effect of Ultrasound-Assisted Sodium Bicarbonate Treatment on Aggregation and Conformation of Reduced-Salt Pork Myofibrillar Protein. Molecules.

[11] Zhu D.Y., Kang Z.L., Ma H.J., Xu X.L., Zhou G.H. (2018). Effect of sodium chloride or sodium bicarbonate in the chicken batters: A physico-chemical and Raman spectroscopy study. Food Hydrocoll..

[12] Li Y.P., Kang Z.L., Sukmanov V., Ma H.J. (2021). Effects of soy protein isolate on gel properties and water holding capacity of low-salt pork myofibrillar protein under high pressure processing. Meat Sci..

[13] Kang Z.L., Zhang X.H., Li K., Li Y.P., Lu F., Ma H.J., Song Z.J., Zhao S.M., Zhu M.M. (2021). Effects of sodium bicarbonate on the gel properties, water distribution and mobility of low-salt pork batters. LWT-Food Sci. Technol..

[14] Zou X., Kang Z.L., Li Y.P., Ma H.J. (2022). Effect of sodium bicarbonate on solubility, conformation and emulsion properties of pale, soft and exudative meat myofibrillar proteins. LWT-Food Sci. Technol..

[15] Zhou F., Zhao M., Zhao H., Sun W., Cui C. (2014). Effects of oxidative modification on gel properties of isolated porcine myofibrillar protein by peroxyl radicals. Meat Sci..

[16] Salvador P., Toldrà M., Saguer E., Carretero C., Parés D. (2009). Microstructure-function relationships of heat-induced gels of porcine haemoglobin. Food Hydrocoll..

[17] Bertram H.C., Kristensen M., Andersen H.J. (2004). Functionality of myofibrillar proteins as affected by pH, ionic strength and heat treatment-a low-field NMR study. Meat Sci..

[18] Liu R., Zhao S.M., Xiong S.B., Xie B.J., Qin L.H. (2008). Role of secondary structures in the gelation of porcine myosin at different pH values. Meat Sci..

[19] Zou Y., Shi H., Xu P., Jiang D., Zhang X., Xu W., Wang D. (2019). Combined effect of ultrasound and sodium bicarbonate marination on chicken breast tenderness and its molecular mechanism. Ult. Sonochem..

[20] Petracci M., Laghi L., Rimini S., Rocculi P., Capozzi F., Cavani C. (2014). Chicken Breast Meat Marinated with Increasing Levels of Sodium Bicarbonate. J. Poult. Sci..

[21] Wachirasiri K., Wanlapa S., Uttapap D., Puttanlek C., Rungsardthong V. (2016). Changing in processing yield and physical properties of frozen white shrimp (*Penaeus vannamei*) treated with lysine and sodium bicarbonate. Int. J. Food Sci. Technol..

[22] Alvarado C., Sams A. (2003). Injection marination strategies for remediation of pale, exudative broiler breast meat. Poult. Sci..

[23] Sen A.R., Naveena B.M., Muthukumar M., Babji Y., Murthy T.R.K. (2003). Effect of chilling, polyphosphate and bicarbonate on quality characteristics of broiler breast meat. Br. Poult. Sci..

[24] Wangtueai S., Noomhorm A., Regenstein J.M. (2010). Effect of Microbial Transglutaminase on Gel Properties and Film Characteristics of Gelatin from Lizardfish (*Saurida* spp.) Scales. J. Food Sci..

[25] Bertram H.C., Meyer R.L., Wu Z., Zhou X., Andersen H.J. (2008). Water Distribution and Microstructure in Enhanced Pork. J. Agric. Food Chem..

[26] Zhang Z., Yang Y., Zhou P., Zhang X., Wang J. (2017). Effects of high pressure modification on conformation and gelation properties of myofibrillar protein. Food Chem..

[27] Tobin B.D., O’Sullivan M.G., Hamill R.M., Kerry J.P. (2012). Effect of Varying Salt and Fat Levels on the Sensory and Physiochemical Quality of Frankfurters. Meat Sci..

[28] Li K., Fu L., Zhao Y.Y., Xue S.W., Wang P., Xu X.L., Bai Y.H. (2020). Use of high-intensity ultrasound to improve emulsifying properties of chicken myofibrillar protein and enhance the rheological properties and stability of the emulsion. Food Hydrocoll..

[29] Zhuang X., Zhang W., Liu R., Liu Y., Xing L., Han M., Kang Z.L., Xu X.L., Zhou G.H. (2017). Improved gel functionality of myofibrillar proteins incorporation with sugarcane dietary fiber. Food Res. Int..

[30] Kang Z.L., Zhu D., Li B., Ma H., Song Z. (2017). Effect of pre-emulsified sesame oil on physical-chemical and rheological properties of pork batters. Food Sci. Technol. Int..

[31] Han M., Zhang Y., Fei Y., Xu X., Zhou G. (2009). Effect of microbial transglutaminase on NMR relaxometry and microstructure of pork myofibrillar protein gel. Eur. Food Res. Technol..

[32] Guo J., Zhou Y., Yang K., Yin X., Ma J., Li Z., Sun W., Han M. (2019). Effect of low-frequency magnetic field on the gel properties of pork myofibrillar proteins. Food Chem..

[33] Chan J.T.Y., Omana D.A., Betti M. (2011). Application of high pressure processing to improve the functional properties of pale, soft, and exudative (PSE)-like turkey meat. Innov. Food Sci. Emerg. Technol..

[34] Mohan A., Jaico T., Kerr W., Singh R. (2016). Functional properties of bicarbonates on physicochemical attributes of ground beef. LWT-Food Sci. Technol..

[35] Kang Z.L., Chen F.S., Ma H.J. (2016). Effect of pre-emulsified soy oil with soy protein isolate in frankfurters: A physical-chemical and Raman spectroscopy study. LWT-Food Sci. Technol..

[36] Kang Z.L., Bai R., Lu F., Zhang T., Gao Z., Zhao S.M., Zhu M.M., Ma H.J. (2022). Effects of high pressure homogenization on the solubility, foaming, and gel properties of soy 11S globulin. Food Hydrocoll..

